# Seroprevalence of mucosal and cutaneous human papillomavirus (HPV) types among children and adolescents in the general population in Germany

**DOI:** 10.1186/s12879-022-07028-8

**Published:** 2022-01-10

**Authors:** Anna Loenenbach, Michael Pawlita, Tim Waterboer, Thomas Harder, Christina Poethko-Müller, Michael Thamm, Raskit Lachmann, Yvonne Deleré, Ole Wichmann, Miriam Wiese-Posselt

**Affiliations:** 1 Department for Infectious Disease Epidemiology, Immunization Unit, Robert Koch-Institute, Berlin, Germany; 2 Charité – Universitätsmedizin Berlin, corporate member of Freie Universität Berlin and Humboldt- Universität zu Berlin, Berlin, Germany; 3 Infections and Cancer Epidemiology, German Cancer Research Center (DKFZ), Heidelberg, Germany; 4 Department of Epidemiology and Health Monitoring, Robert Koch-Institute, Berlin, Germany; 5 GP Practice, Berlin, Germany; 6 Institute of Hygiene and Environmental Medicine, Charité – Universitätsmedizin Berlin, corporate member of Freie Universität Berlin and Humboldt- Universität zu Berlin, Berlin, Germany

**Keywords:** Human papillomavirus, Seroprevalence, Risk factors, Children, Adolescents, Germany

## Abstract

**Background:**

In Germany, HPV vaccination of adolescent girls was introduced in 2007. Nationally representative data on the distribution of vaccine-relevant HPV types in the pre-vaccination era are, however, only available for the adult population. To obtain data in children and adolescents, we assessed the prevalence and determinants of serological response to 16 different HPV types in a representative sample of 12,257 boys and girls aged 1–17 years living in Germany in 2003–2005.

**Methods:**

Serum samples were tested for antibodies to nine mucosal and seven cutaneous HPV types. The samples had been collected during the nationally representative German Health Interview and Examination Survey for Children and Adolescents in 2003–2006. We calculated age- and gender-specific HPV seroprevalence. We used multivariable regression models to identify associations between demographic and behavioral characteristics and HPV seropositivity.

**Results:**

We found low but non-zero seroprevalence for the majority of tested HPV types among children and adolescents in Germany. The overall seroprevalence of HPV-16 was 2.6%, with slightly higher values in adolescents. Seroprevalence of all mucosal types but HPV-6 ranged from 0.6% for HPV-33, to 6.4% for HPV-31 and did not differ by gender. We found high overall seroprevalence for HPV-6 with 24.8%. Cutaneous HPV type seroprevalence ranged from 4.0% for HPV-38 to 31.7% for HPV-1. In the majority of cutaneous types, seroprevalence did not differ between boys and girls, but increased sharply with age, (e.g., HPV-1 from 1.5% in 1–3-years-old to 45.1% in 10–11-years-old). Associations between behavioral factors and type-specific HPV prevalence were determined to be heterogeneous.

**Conclusions:**

We report the first nationally representative data of naturally acquired HPV antibody reactivity in the pre-HPV-vaccination era among children and adolescents living in Germany. These data can be used as baseline estimates for evaluating the impact of the current HPV vaccination strategy targeting 9–14-years-old boys and girls.

**Supplementary Information:**

The online version contains supplementary material available at 10.1186/s12879-022-07028-8.

## Background

Infections with human papillomaviruses (HPVs) are among the most common sexually transmitted infections but can also be transmitted perinatally. HPV belongs to the diverse Papillomaviridae virus family and comprises over 200 different types, which can be further categorized based on different parameters [1–3]. HPV types belong to different genera and species based on their phylogenetic relationship [4]. They can also be categorized into two tropism groups, comprising cutaneous HPV types (cutHPV) and mucosal types (mucHPV). Only a small fraction of mucHPV belonging to the alpha genus, like HPV-16 and HPV-18, are assigned to the so-called high-risk (HR) group, due to their role as causative agents of various types of precancerous lesions and cancer [5, 6]. However, the majority of HPV-infected persons develop no visible signs or symptoms, and infections are usually transient and cleared within 12–24 months [5, 7–9]. Infections with other HPV types can lead to different clinical manifestations. The so-called low-risk (LR) types HPV-6 and HPV-11 can cause external benign genital warts [6]. CutHPV, e.g. HPV-1 or HPV-4, are usually found on healthy skin [10], even though they can also be detected in skin lesions such as benign skin warts [4, 6], and some types have been discussed to be involved in skin carcinogenesis [11].

Children and adolescents before sexual debut can also be affected by HPV infections. Typical disease manifestations of HPV infection in children are skin warts [12], commonly transmitted by cutHPV infections [13]. Skin warts are mainly caused by HPV types 1, 2, 3, 4, 27 and 57 [3]. There are different types of warts, including common warts (verruca vulgaris), plantar warts (myrmecias) and flat warts (verruca plana) with different prevalence and age distributions, which could be due to differences in transmission modes [3, 14]. Generally, HPV associated skin warts are rare in preschool children, and peak among children aged 10–14 years, followed by a rapid decline at 20 years of age with no difference between girls and boys [3]. Other rare diseases caused by HPV infections in children and adolescents are juvenile-onset recurrent respiratory papillomatosis (JoRRP), oral papilloma or anogenital warts [15]. JoRRP is most prevalent among children under five, mainly caused by persistent HPV-6 and HPV-11 infections and associated with maternal transmission [15]. However, there is also growing evidence of HR-HPV infections in healthy children [3, 16].

There are only few HPV serology data in children available, even though research on HPV infections in children already started more than 50 years ago [17–19]. Research primarily focused on modes of HPV transmission such as perinatal mother-infant transmission [16] or prevalence among adolescents related to the start of sexual activities. Study results on risk of vertical transmission vary [20, 21] and the exact routes remain unclear [22]. Nevertheless, detection of genital HR-HPV DNA in infants has been repeatedly reported by various studies [21, 23, 24]. Next to perinatal transmission, alternative routes were addressed, such as periconceptual, antenatal, via amniotic fluid [22]. HPV infections can be also horizontally transmitted via autoinoculation, heteroinoculation or via fomites [3, 25, 26]. HPV infections leading to anogenital warts in children are also discussed as a result of sexual transmission in the context of sexual abuse [14, 27]. Anogenital warts, based on vertical or sexual transmission are mainly due to mucHPV 6, 11, 16 und 18 [3, 27]. Anogenital warts due to cutHPV 1, 2, 3 or 4 can be due to hetero- or autoinoculation [3]. It is important to notice, however, that the exact transmission routes in infants and children remain controversial [3, 26, 28], which makes it even more relevant to evaluate age-specific prevalence data in this population.

To investigate the prevalence of HPV infections, DNA testing is used as the reference standard for the detection of current HPV infections [29]. While DNA testing is not an appropriate method to assess previous infections [30], testing for HPV-specific antibodies in an unvaccinated population provides information about past HPV exposure [30, 31]. HPV serology has been established as an important method for population-based studies focusing on type-specific cumulative lifetime exposure to HPV. However, there are only few seroprevalence studies that focus on children [16, 32–34].

Our study aimed to determine age-specific HPV seroprevalence in a representative sample of 12,257 boys and girls aged 1–17 years living in Germany in the years 2003 to 2005. As HPV vaccination of girls has only been introduced in Germany in 2007, our results show naturally acquired antibody reactivity in the pre-HPV vaccination era.

## Methods

### Study population

Archived serum samples were obtained from the German Health Survey for Children and Adolescents (KiGGS) carried out by the Robert Koch Institute (RKI) from 2003 to 2006 [35]. This cross-sectional health survey was the baseline study of the RKI health monitoring program and aimed to collect and analyze nation-wide representative data on the health status of children and adolescents in Germany aged 0–17 years [36]. Recruitment was based on a two-stage stratified cluster sampling design, with participants randomly selected from the population registers of 167 cities in Germany (for a detailed description of the study design, see [36, 37]). The overall response rate was 66.6% [36].

The study was conducted according to the Federal and State Commissioners for Data Protection guidelines and was approved by the Charité University Medicine Berlin ethics committee and the Federal Commissioner for Data Protection. Informed written consent and agreement were obtained from the parents of all participants.

### Survey methods

In the KiGGS study, a total of 17,641 children and adolescents aged 0–17 years were interviewed and medically examined. Of those, 14,386 participants aged 1–17 years provided a blood sample. As we could not test around 15% of the sera samples because they were already used up in the core study (n = 14,302), or could not be successfully tested in the laboratory for reasons like insufficient bead count (n = 84), the overall number of serum samples available for this study were 12,257 (85.2%).

Standardized self-administered questionnaires were used to obtain information on socio-demographic and lifestyle variables. Questionnaires were filled out by the parents of all children (1–17 years) and by the children themselves (> 10 years). Age was categorized into seven groups (1–3, 4–6, 7–9, 10–11, 12–13, 14–15, and 16–17). Region of residence was split into two groups, with ‘West Germany’ and ‘East Germany’, considering the former boarders of the German Democratic Republic and the Federal Republic of Germany from 1949 to 1990. Urbanity was categorized as rural (< 5000 residents), small city (5000 to < 20,000 residents), medium-sized city (20,000 to < 100,000 residents) and large city (≥ 100,000 residents). The socioeconomic status was based on a parental socioeconomic status (SES) index, including information about education, occupational status, and income of both parents separately. The highest index score was used for the overall household SES. Based on the household SES index, children were categorized into ‘low’, ‘medium’ and ‘high’ SES [38].

### Multiplex serology

In 2016/2017, serum specimens were tested for antibodies to the major capsid (L1) protein of 16 different HPV genotypes at the German Cancer Research Center (DKFZ) in Heidelberg. Serological testing was performed by a glutathione *S*-transferase (GST) capture immunoassay in combination with fluorescent bead technology as previously described [39]. We used the following criteria to select HPV-types for analysis: public health relevance, carcinogenic potential, and associated disease outcomes (Additional file 1: Fig. S1). As little is known about different cutaneous HPV types in children apart from the common cutaneous disease types HPV-1 and HPV-4, we tried to include a broad coverage of phylogenetic genera and species for cutHPV. Finally, nine mucosal (alpha: 6, 11, 16, 18, 31, 33, 45, 52, 58) and seven cutaneous (alpha: 10; beta: 8, 38, 49; gamma: 4; nu: 41; mu: 1) HPV genotypes were included in the test panel. We measured type-specific HPV seroreactivity in median fluorescence intensity (MFI) units.

For calculating seropositivity, MFI values were dichotomized as positive or negative based on previously established type-specific cutoff-values. Seropositivity was defined as the proportion (%) of positive tested sera. The cut-off values were established with sera of a cohort of 125 young Korean women who were HPV DNA negative and self-reported to never have had sexual intercourse [40]. The following MFI cutoffs were used for mucHPV: HPV-6: 571, HPV-11: 500, HPV-16: 200, HPV-18: 200, HPV-31: 712, HPV-33: 515, HPV-45: 368, HPV-52: 371, HPV-58: 200. A cutoff of 200 MFI was used for all cutHPV [41].

### Statistical analysis

To assure representativeness of the data at the national level, survey weights were calculated and applied to all estimates, adopting the study sample (providing HPV antibody test results) to the population structure of Germany in 2003 in terms of age, gender, state, size of municipality, education and German/non-German nationality and the regional distribution between East and West Germany. These survey weights, which accounted for the stratified and clustered sample design of the survey, were applied throughout the statistical analyses.

Weighted seroprevalence was calculated for all 16 types separately and for the following groups of HPV types: types included in the bivalent vaccine (HPV-2val, 16, 18), types included in the quadrivalent vaccine (HPV-4val, 6, 11, 16, 18), and types included in the nonavalent vaccine (HPV-9val, 6, 11, 16, 18, 31, 33, 45, 52, 58). Group-specific seroprevalence was calculated as the weighted proportion of participants seropositive to at least one of the HPV-types included in one group. Additionally, MFI were plotted against the percentile for each HPV type individually stratified by gender and age for analyzing antibody reactions without relying on a specific cutoff.

Differences regarding demographic and behavioral characteristic of participants stratified by gender were evaluated by using chi^2^ or Fishers Exact test. Chi^2^-tests were used to test for statistical significance in categorical variables (p < 0.05) and logit transformation was applied to calculate confidence intervals (95% CI).

We calculated prevalence ratios (PRs) using Poisson regression models to identify factors independently associated with HPV seropositivity for HPV-6, HPV-11, HPV-16, HPV-18 and for at least one of the cutaneous HPV types (HPV-1, HPV-4, HPV-8, HPV-10, HPV-38, HPV-41, or HPV-49) tested for in our analysis (HPV-cut). We used PR instead of Odds Ratios to obtain more interpretable association estimates [42]. The modelling was performed using generalized linear models with Poisson family with log link function. Additionally, we included the survey design for estimating variance and 95% CIs. Possible interactions between factors were taken into consideration in the multivariable model. In the final multivariable model, we included all factors that were associated with type-specific seropositivity at a p < 0.05 level in a backward step approach. Pearson’s correlation coefficient was calculated to identify correlations between HPV types, with attributing small correlation for values between 0.1 and 0.3, moderate correlation for values between 0.3 and 0.5 for strong correlation for values 0.5.

Data management and statistical analysis were conducted using Stata, Version 14 (STATA Corp., College Station, TX, US). Percentile plots were created with R Studio, Version R version 3.6.0 (2019-04-26).

## Results

**Fig. 1:**
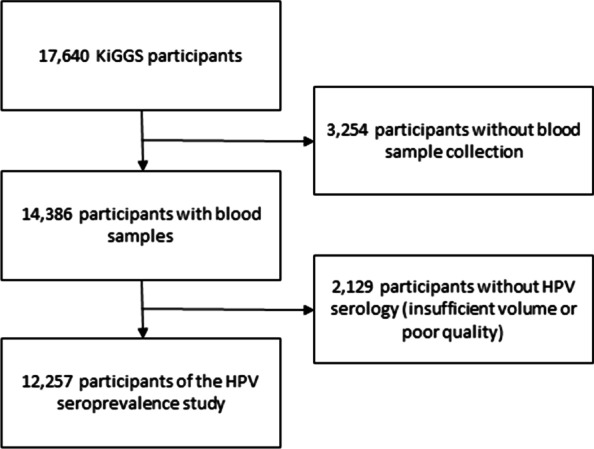
Flow chart of study participants of the HPV seroprevalence study (n = 12,257, sera collected 2003–2006)

Overall, 12,257 serum samples of children and adolescents aged 1–17 years with valid HPV serology from the pre-HPV-vaccination era were included in the analysis (Fig. 1). Among the participants, 48.7% were girls (n = 5973) and 51.3% were boys (n = 6284). The sociodemographic characteristics of the study population are shown in Table 1.

**Table 1:** Demographic characteristics of the study participants stratified by gender, HPV seroprevalence study (n = 12,257, sera collected 2003–2006)

	Females		Males		Total		p-value
	Subjects, no.	%^a^	Subjects, no.	%^a^	Subjects, no.	%^a^	
Overall	5973	48.7	6284	51.3	12,257	100	
Age group (y)							0.248
1–3	616	15.4	649	15.4	1265	15.4	
4–6	882	16.7	933	16.6	1815	16.6	
7–9	1071	17.0	1146	17.0	2217	17.0	
10–11	823	11.4	851	11.4	1674	11.4	
12–13	889	12.2	960	12.2	1849	12.2	
14–15	858	13.6	963	13.6	1821	13.6	
16–17	834	13.8	782	13.8	1616	13.8	
Region of residence							0.426
West Germany	3922	83.2	4169	83.2	8091	83.2	
East Germany	2051	16.8	2115	16.8	4166	16.8	
Urbanity							0.090
Rural	1338	18.3	1382	18.5	2720	18.4	
Small city	1561	27.5	1660	27.4	3221	27.5	
Medium sized city	1768	29.6	1764	29.1	3532	29.3	
Large city	1306	24.6	1478	25.0	2784	24.8	
Socioeconomic status of parents							0.612
Low	1569	33.8	1656	34.3	3225	34.1	
Middle	2744	43.0	2882	42.0	5626	42.5	
High	1523	20.9	1579	21.1	3102	21.0	
NA	137	2.4	167	2.6	304	2.5	
Migratory background of parents							0.319
None	4680	74.2	4891	74.0	9571	74.1	
One parent	403	8.1	431	8.4	834	8.3	
Both parents	861	17.3	943	17.3	1804	17.3	
NA	29	0.5	19	0.3	48	0.4	

*NA* not available

^a^Weighted proportion

^#^p-value for difference by gender

### Seroprevalence mucosal HPV types

**Fig. 2:**
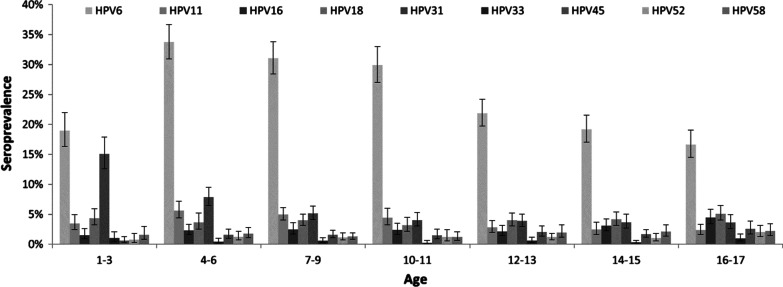
Seroprevalence of mucosal human papillomavirus types by age, HPV seroprevalence study (n = 12,257, sera collected 2003–2006)

Seroprevalence data and MFI distributions of mucHPV are presented in Fig. 2, and Additional file 2: Fig. S2, Additional file 5: Table S1. Seroprevalence of all but HPV-6 ranged from 0.6% (95% CI 0.4–0.8%) for HPV-33, to 6.4% (95% CI 5.8–7.1%) for HPV-31. Type-specific HPV seroprevalence did not differ by gender for most mucHPV, except of HPV-6.

**HPV-6** differed to the other mucHPV with a high overall seroprevalence of 24.8% (95% CI 23.6–26.1%). The highest value (33.8%, 95% CI 31.0–36.7%) was observed in the age group 4–6 years. HPV-6 seroprevalence decreased thereafter to 16.6% (95% CI 14.5–19.1%) in the age group 16–17 years. HPV-6 was the only mucHPV which differed by gender with an overall seroprevalence of 23.1% (95% CI 21.5–24.7%) in girls and 26.4% (95% CI 24.9–28.0%) in boys (p < 0.001). HPV-11 resembled HPV-6 regarding age distribution with highest seroprevalence in the age group 4–6 years.

The overall seroprevalence of **HPV**-**16** was 2.6% (95% CI 2.2–3.0%). It remained relatively stable in the youngest age groups with 1.5% (95% CI 0.9–2.6%) among the 1–3 years-old and 2.1% (95% CI 1.4–3.1%) among the 12–13 years-old and increased thereafter to 3.1% (95% CI 2.2–4.2%) and 4.4% (95% CI 3.4–5.8%) among the age groups 14–15 and 16–17 years, respectively.

**HPV-18** seroprevalence had a low variability across age groups, with highest seroprevalence in the oldest group (5.1%, 95% CI 4.0–6.4%). Overall, lowest seroprevalence was found for HPV types 33, 45, 52, and 58, with slightly higher values in older age groups. HPV-31 showed the highest seroprevalence (15.1%, 95% CI 12.6–17.9%) in the youngest age group, strongly decreasing at higher age.

### Seroprevalence of vaccine relevant HPV types

Overall, 6.1% (95% CI 5.5–6.8%) of the participants were seropositive for at least one of HPV-16 or HPV-18, both types targeted by the bivalent vaccine (HPV-2val) (data not shown). 28.9% (95% CI 27.5–30.4%) were seropositive for at least one of the types covered by the quadrivalent vaccine (HPV-4 val) and around a third (34.0%, 95% CI 32.5–35.6%) were seropositive for one of the nonavalent vaccine types (HPV-9val).

### Seroprevalence of cutaneous HPV types

**Fig. 3:**
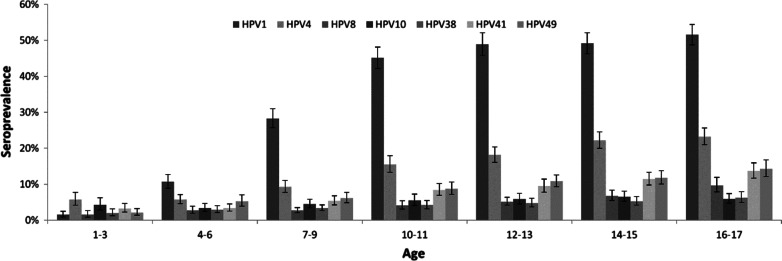
Seroprevalence of seven cutaneous human papillomavirus types by age, HPV seroprevalence study (n = 12,257, sera collected 2003–2006)

Seroprevalence data and MFI distributions of cutHPV are presented in Fig. 3, Additional file 3: Fig. S3 and Additional file 6: Table S2. CutHPV seroprevalence ranged from 4.0% (95% CI 3.5–4.6%) in girls and 4.0% (95% CI 3.5–4.5%) in boys for HPV-38 to 33.8% (95% CI 32.2–35.5%) in girls and 29.7% (95% CI 28.4–31.1%) in boys for HPV-1 (Additional file 6: Table S2). In most cutHPV, HPV seroprevalence did not differ between boys and girls, except of HPV-1 with 33.8% (95% CI 32.2–35.5%; girls) and 29.7% (95% CI 28.4–31.1% boys) (p < 0.001).

CutHPV seroprevalence increased nearly steadily from youngest to oldest age groups. We found the strongest increase in HPV-1 seroprevalence, ranging from 2.1% (95% CI 1.1–4.0%) in 1–3-years-old to 55.7% (95% CI 51.7–59.6%) in 16–17-years-old among girls and from 0.9% (95% CI 0.4–1.8%) in 1–3-years-old to 47.7% (95% CI 43.4–52.0%) in 16–17-years-old among boys.

Taken all seven tested cutaneous HPV types together, 46.0% (95% CI 44.7–47.3%) of the participants were seropositive for at least one HPV-cut type (data not shown). Seroprevalence for HPV-cut increased sharply from 16.0% (95% CI 13.5–18.9%) in the age group 1–3 years to 57.5% (95% CI 54.4–60.6%) among children aged 10–11 years. Thereafter, seroprevalence for HPV-cut remained relatively stable and increased only slightly to 66.9% (95% CI 64.2–69.5%) in age group 16–17. No seroprevalence difference was observed between females and males in overall HPV-cut (p = 0.087).

### Seroprevalence of single or multiple HPV types

36.6% (95% CI 35.1–38.2%) of the study population was seronegative for all 16 investigated HPV types. Around a third (32.7%, 95% CI 31.5–34.0%) were seropositive for one HPV type, and 16.8% (95% CI 15.8–17.7%) were seropositive for two HPV types. 7.4% (95% CI 6.7–8.2%) of the study population were seropositive for three HPV types, and 6.5% (95% CI 5.9–7.1%) were seropositive for more than three HPV types. In general, multiple HPV seropositivity was influenced by the high proportion of HPV-6 seropositivity. No gender difference was observed for multiple HPV seropositivity (p = 0.475).

The percentage of children being seronegative to any HPV types decreased from 57.2% (95% CI 53.1–61.1%) among 1–3-years-old to 28.8% (95% CI 26.1–31.6%) among 10–11-years-old children and decreased only slightly thereafter to 25.7% (95% CI 23.3–28.3%) among the oldest age group (16–17 years) (Additional file 4: Fig. S4). Accordingly, seropositivity for three or more HPV types increased from 4.7% (95% CI 3.6–6.2%) with 3 types and 1.6% (95% CI 0.9–2.8%) with > 3 types among the youngest to 9.6% (95% CI 8.1–11.3%) with 3 types and 12.4% (95% CI 10.3–14.7%) with > 3 types among the oldest age group.

Strong correlation of MFI values was observed among HPV α10 genotypes 6 and 11 (r = 0.85) and HPV α9 types 52 and 58 (r = 0.75). Moderate correlation was observed among α7 types 18 and 45 (r = 0.46) and among α9 types, with HPV types 31/33 (r = 0.39), 33/52 (r = 0.49), and 33/58 (r = 0.49).

Regarding cutHPV, moderate correlation was found only between HPV types 8 and 38 (r = 0.44), 8 and 49 (r = 0.47), and 38 and 49 (r = 0.43).

### Factors associated with type-specific seropositivity

We analyzed possible associations of demographic variables with type-specific seropositivity. The weighted crude and adjusted PR for HPV-16 and HPV-cut can be found in Tables 2 and 3, respectively. Results of the regression analysis for HPV-6, HPV-11, and HPV-18 seropositivity are presented in Additional file 7: Table S3, Additional file 8: Table S4 and Additional file 9: Table S5.

**Table 2:** Factors associated with seropositivity for HPV-16, HPV seroprevalence study (n = 12,257, sera collected 2003–2006) (results from regression analysis)

	Crude PR (95% CI)	p-value	Fully adjusted PR (95% CI)^a^	p-value
Gender			ns^b^	
Female	Ref			
Male	1.1 (0.8–1.4)	0.706		
Age group (years)				
1–3	Ref		Ref	
4–6	1.5 (0.8–3.0)	0.196	1.5 (0.8–3.0)	0.198
7–9	1.6 (0.8–3.3)	0.158	1.6 (0.8–3.2)	0.167
10–11	1.6 (0.8–3.2)	0.184	1.6 (0.8–3.1)	0.195
12–13	1.4 (0.7–2.8)	0.331	1.4 (0.7–2.8)	0.348
14–15	2.0 (1.1–3.7)	0.018	2.1 (1.2–3.7)	0.015
16–17	3.0 (1.6–5.5)	0.001	3.0 (1.6–5.6)	0.001
Region of residence				
West Germany	Ref		Ref	
East Germany	0.7 (0.5–1.0)	0.055	0.7 (0.5–1.0)	0.025
Urbanity			ns^b^	
Rural	Ref			
Small city	0.8 (0.6–1.3)	0.423		
Medium sized city	0.9 (0.6–1.3)	0.499		
Large city	0.8 (0.5–1.3)	0.370		
Socioeconomic status of parents			ns^b^	
Low	Ref			
Middle	0.8 (0.6–1.1)	0.132		
High	0.8 (0.5–1.1)	0.134		
Migratory background of parents			ns^b^	
None	Ref			
One parent	0.9 (0.5–1.5)	0.596		
Both parents	1.3 (1.0–1.9)	0.077		
Number of household members	1.0 (0.9–1.1)	0.752	ns^b^	
Number of siblings in household	0.9 (0.8–1.1)	0.225		
Body Mass Index (BMI)	1.0 (1.0–1.1)	< 0.001	ns^b^	

*PR* prevalence ratio, *CI* confidence interval, *Ref* reference

^a^Mutually adjusted for all other variables in the model

^b^ns = variables were not significantly associated with HPV seroprevalence in the final model and therefore excluded

**Table 3:** Factors associated with seropositivity for HPV-cut, HPV seroprevalence study (n = 12,257, sera collected 2003–2006) (results from regression analysis)

	Crude PR (95% CI)	p-value	Fully adjusted PR (95% CI)^a^	p-value
Gender			ns^b^	
Female	Ref			
Male	1.0 (0.9–1.0)	0.086		
Age group (y)				
1–3	Ref		Ref	
4–6	1.6 (1.3–1.9)	< 0.001	1.6 (1.3–1.9)	< 0.001
7–9	2.6 (2.2–3.1)	< 0.001	2.6 (2.2–3.1)	< 0.001
10–11	3.6 (3.0–4.3)	< 0.001	3.6 (3.0–4.3)	< 0.001
12–13	4.0 (3.3–4.7)	< 0.001	3.9 (3.3–4.7)	< 0.001
14–15	4.0 (3.4–4.7)	< 0.001	4.0 (3.4–4.7)	< 0.001
16–17	4.2 (3.5–5.0)	< 0.001	4.2 (3.5–5.0)	< 0.001
Region of residence				
West	Ref		Ref	
East	1.0 (0.9–1.0)	0.084	0.9 (0.9–1.0)	< 0.001
Urbanity				
Rural	Ref		Ref	
Small city	1.0 (0.9–1.0)	0.433	1.0 (0.9–1.0)	0.447
Medium sized city	0.9 (0.8–1.0)	0.006	0.9 (0.9–1.0)	0.042
Large city	0.9 (0.8–0.9)	0.001	0.9 (0.9–1.0)	0.045
Socioeconomic status of parents			ns^b^	
Low	Ref			
Middle	1.0 (1.0–1.1)	0.211		
High	1.0 (0.9–1.0)	0.329		
Migratory background of parents				
None	Ref		Ref	
One parent	0.9 (0.8–1.0)	0.018	1.0 (0.9–1.1)	0.896
Both parents	0.9 (0.8–0.9)	< 0.001	0.9 (0.8–0.9)	0.001
Number of household members	1.0 (1.0–1.1)	0.003	ns^b^	
Number of siblings in household	1.0 (1.0–1.1)	< 0.001	ns^b^	
BMI	1.1 (1.0–1.1)	< 0.001	ns^b^	
Breastfeeding			ns^b^	
Never	Ref			
Yes (but not solely)	1.0 (1.0–1.1)	0.345		
Yes, full till 4th month	0.9 (0.9–1.0)	0.033		
Yes, full till 6th month	0.8 (0.8–0.9)	< 0.001		
Sunburn			ns^b^	
No	Ref			
Yes, several times	1.4 (1.2–1.5)	< 0.001		
Yes, one time	1.4 (1.3–1.4)	< 0.001		
Don't know	1.3 (1.2–1.5)	< 0.001		

*PR* prevalence ratio, *CI* confidence interval, *Ref* reference

^a^Mutually adjusted for all other variables in the model

^b^ns = variables were not significantly associated with HPV seroprevalence in the final model and therefore excluded

In the fully adjusted **HPV-16 model**, only age and region of residence were significantly associated with seropositivity. Children of older age (PR 14–15 years: 2.1, 95% CI 1.2–3.7; 16–17 years: 3.0, 95% CI 1.6–5.6) and children living in West Germany (PR: 0.7, 95% CI 0.5–1.0) were more likely to be seropositive compared to younger age groups and to those living in the eastern part of Germany.

While several associations, like number of household members, or number of sunburns, were significant in the univariable **HPV-cut model**, a significant association with HPV-cut seropositivity was only observed for age, region of residence, urbanity, and migratory background of parents in the multivariable model. The strongest association with HPV seropositivity for cutHPV was seen for age, with a PR increase from 1.6 (95% CI 1.3–1.9) among the youngest age groups to 4.2 (95% CI 3.5–5.0) among the oldest. Living in East Germany, compared to West Germany, was associated with a slightly lower seropositivity (0.9, 95% CI 0.9–1.0). The same was true for living in medium (0.9, 95% CI 0.9–1.0) or large cities (0.9, 95% CI 0.9–1.0), compared to living in rural areas, as well as for migratory background of parents (0.9, 95% CI 0.8–0.9), compared to non-migratory background.

Region of residence was the only variable which was significantly associated with HPV seropositivity in all five regression models. Children living in West Germany were more likely to be seropositive for HPV-6, HPV-11, HPV-16, HPV-18, and HPV-cut, compared to children living in East Germany. HPV-6 and HPV-11 were the only serotypes for which number of siblings (HPV-6: 1.1, 95% CI 1.1–1.1) or number of household members (HPV-11: 1.1, 95% CI 1.0–1.2) were significantly associated with a slightly higher seropositivity (Additional file 7: Table S3, Additional file 8: Table S4).

## Discussion

Data on HPV infections from the pre-vaccination era are crucial to evaluate the impact of HPV vaccination. Although not all HPV infections lead to seroconversion, population-based seroprevalence studies are suitable to inform about prior cumulative exposure to HPV in different age groups. While most of the serological studies focus on adult population or adolescents, data on type-specific antibody reactivity in children and adolescents of all ages and both genders are scarce. We examined nationally representative data on children and adolescents aged 1–17 years to determine seroprevalence of nine mucosal and seven cutaneous HPV serotypes in 2003 to 2006, before the introduction of HPV vaccines in Germany.

HPV seroprevalence of mucHPV was generally low among children and adolescents. Type-specific seroprevalence was < 3% for HPV-16, 33, 45, 52, and 58, around 4% for HPV-18 and 11, and around 6% for HPV-31. HPV-6 showed relatively high antibody reactivity with around a quarter of the children and adolescents being seropositive. We observed considerable seroprevalences of several mucHPV in children who were above the age of having maternal antibodies gained through a potential vertical transmission and under the age of being sexually active and having gained HPV antibodies through sexual contact. However, most of the children showed low type-specific mucosal antibody titers, compared to cutHPV. Seroprevalence of cutHPV were generally higher, with most HPV types ranging between 4 and 8%, as well as 14% and 32% for HPV-4 and HPV-1, respectively. High reactivity in cutHPV was especially evident for HPV-1, which was present in around 50% of the children above the age of 10 years.

While many international studies have investigated HPV serology in adults, only few serological studies targeted children and adolescents [33, 43–50]. However, most of them are limited by small sample size [41, 49, 51, 52], focus on single or highly selected mucHPV [32, 44, 47], or target specific age groups [50, 53]. Most serological studies do not analyze age-specific seroprevalence of children under the age of 10 (mainly because of small sample size) [41, 43, 45, 51, 52, 54–56], or include only females [45, 57, 58].

The focus on HPV prevalence among infants or adolescents is usually based on different theories and approaches of HPV transmission in children and adolescents. Mainly older studies focused on exploring seroprevalence based on vertical (mainly perinatal) transmission, reporting varying risk of transmission between 4 and 22%. Maternal transmission was addressed by several research papers [16, 20, 21, 27, 59], showing that about 30% of HPV positive children share at least one (cutaneous) HPV type with their mother (or also father) [3]. Although perinatally acquired HPV infections may persist up to 3 years [24, 60, 61], it was concluded that the overall risk of vertical HPV transmission is relatively low [62].

Others have analyzed the age-dependent increase of mucosal HPV seroprevalence in older children and adolescents which was assumed to reflect sexual transmission of HPV due to onset of sexual activity. However, mucosal HPV prevalence was also detected in children of other age groups, irrespective of sexual transmission [16, 18]. Subsequently, it has been increasingly described that mucosal antibody reactions (or HPV DNA detection) in children should not be automatically seen as a reliable marker of sexual abuse and alternative transmission routes of HPV should be considered [3, 20, 27, 56, 63, 64]. Non-sexual and non-vertical HPV infections in children have been described as potentially horizontally transmitted [20], by self- or heteroinoculation (e.g. anogenital to hands or finger to mouth) [3, 16, 65]. This is supported by Syrjanen [63], who describes that typical anogenital HPV types, like HPV 6, 11, or 16, are also commonly found in oral mucosa [66, 67]. The horizontal transmissions may explain the seroprevalence of mucHPV among children in our study. Another explanation is that early acquired infections through vertical transmissions can lead to latent infections in children, which may remain for several years [63, 68].

Type-specific HPV seroprevalence in children showed a considerable variability in previous studies. HPV-16 seroprevalence ranged from 0.0% in a study with 128 children aged 0–9 years in Australia [43] to 10.9% in a study with 46 children aged 2–7 years in South Africa [69]. In a study including 257 male students aged 9–14 years from Mexico City, HPV 16 seroprevalence was 6.2% [70]. A study from Germany including 187 children aged 1–14 years measured a seroprevalence of 0.5% [41]. However, age groups within studies differed substantially and numbers of participants (under the age of 18 years) were small in all studies. A study by Dunne et al. [55], including higher numbers of children, reported an HPV-16 seroprevalence of 2.4% among 1,316 children aged 6–11 years in the United States. They found a seroprevalence of 0.4% among 429 children aged 6–7 and 3.3% among children aged 8–11 years. A study by Cubie and colleagues reported an HPV-16 seroprevalence of 7.6% among 1192 schoolgirls aged 11–13 years, which is higher compared the HPV-16 seroprevalence of 2.7% and 2.1% among girls aged 10–11 and 12–13 years found in our study [50]. A study from Sweden including 1031 children aged 0–13 years calculated an HPV-16 seroprevalence of 3.0% [32], which is comparable to the overall HPV-16 seroprevalence of 2.6% in our study population.

In the same study, HPV-16-seroprevalence was highest (5.2%) among infants aged 0–0.5 years and again high with 6.1% and 3.2% among children aged 7–10 (n = 165) and 10–13 years of age (n = 124) [32]. A bimodal age distribution has been discussed before [18] and was also reported for asymptomatic HPV infections of the oral mucosa, with a first peak prior to 1 year of age and a second peak in adolescence [3]. In our data, HPV-16 seroprevalence remained relatively stable between 1.5 and 2.5% among children under the age of 14 years and increased slightly to 4.4% in the oldest age group, following a rather typical age distribution based on an increased exposure due to sexual transmission of HPV-16 as reported by others [45, 56, 57, 71]. However, it is important to notice, that we could not include infants under the age of 1 year.

An age distribution similar to that of HPV-16 seroprevalence was observed for HPV-18, HPV-45, HPV-52 and HPV-58. The opposite was true for HPV-6, HPV-11, and HPV-31: the highest seroprevalence was observed in younger age groups, followed by lowest HPV seroprevalence in the oldest age groups. This is not in line with results from another study, even though a comparison is limited as younger age groups are combined broadly and numbers are low within those age groups in the study from Australia [43]. However, a bimodal age distribution with high numbers of HPV infections (e.g. HPV-6) among the youngest age groups were also found by DNA testing [72]. Compared to most mucHPV, we observed higher seroprevalence of cutHPV, especially for HPV-1, which is in line with other reports [41, 50].

Using HPV-specific antibodies as a measure of previous infections poses several challenges. While it has been shown that mucosal HPV antibodies can serve as an indicator of previous HPV infections [73], varying seroconversion rates [74] and latency time of antibody development limit their value. In females, following natural infections with HPV, antibody responses are only detectable in about 50–70% of cases [74, 75], with the majority of responses being weak. A study by Antonsson et al. [76] showed that there was little difference in HPV antibody stability between men and women. Another limitation of serological HPV studies is the question of antibody stability over time. Whereas antibody titers have been shown to be relatively stable for mucosal types [73], the stability of cutHPV antibodies is less known [76] and studies with reliable data on the HPV antibody stability among children of different age groups are missing.

It has been discussed whether the development of HPV antibodies (and more generally an HPV infection) in early childhood protects from HPV infection or HPV associated diseases in later life [62, 77, 78]. Naturally acquired HPV antibodies provide protection against subsequent cervical HPV infections [79]. However, the effect of naturally acquired infections on immunity seems to be modest and type-specific, as the effect was only observed for HPV-16 infections [78, 79]. Rodriguez et al. [80] showed that type-specific HPV infections may reappear and may lead to precancerous lesions in previously exposed individuals, even though the risk was low. In addition, the magnitude of antibody response seems to influence immunity as shown in natural acquired compared to vaccination acquired antibody responses [62, 79, 81, 82]. The effect of naturally acquired HPV‐16 antibodies seems to be also age-dependent and was lower in women > 25 years compared to 15‐ to 25‐year‐old women [62]. Focusing on seroprevalence in children and the potential influence of age, the question of naturally acquired immunity is even more relevant when investigating the most appropriate age for vaccination.

In Germany, HPV vaccination is recommended for girls by the Standing Committee on Vaccination (STIKO) since 2007 [83]. In 2018, the recommendation of a two doses HPV vaccine schedule (with an interval of at least 5 month) was extended to all boys and girls at ages 9–14 years [84]. HPV vaccination coverage in Germany is still low but coverage data indicates a steady increase of full (two dose) coverage among 15 year old girls from 27.2% in 2011 to 43.3% in 2018 [85]. Regarding HPV vaccination strategies, it is generally recommended that HPV vaccine should be given to children prior to sexual debut. On the basis of study results showing that children are already exposed to HR HPV types in young ages, it has been argued that prophylactic HPV vaccination could be more beneficial, if given at an earlier time point, e.g. at birth or in early childhood [16].

We observed no difference in HPV seropositivity in children regarding most of the demographic factors, like gender, or socioeconomic status. The significant higher prevalence ratio for HPV-16 seropositivity in the age groups 15–17 years is in line with other publications and an expression of the sexual debut and the increase in sexual contacts. Slightly higher seroprevalence was found in West Germany compared to East Germany both for mucHPV 6, 11, 16 and 18, and for HPV-cut. The regional difference mirrors the old state border between the Federal Republic of Germany and the German Democratic Republic (1949 till 1990). Therefore, it is a potential factor of underlying socio-cultural differences between the two regions and described as a potential factor for prevalence differences of infectious diseases before [86].

There are a few conflicting studies about a potential association between obesity, measured mostly as body mass index, and HPV infections [87–89]. In our data, body mass index was negatively associated with HPV-16 seroprevalence in the univariate model of our data but was not significant in the final model. Even though body mass index may potentially play a role as a psychosocial risk factor in HPV exposure based on its influence on sexual activity [90], our data underline that there is no biological plausibility of such an association as this would have been seen in younger ages as well. We did not find any association between number of household members or number of siblings and HPV-16, HPV-18, or HPV-cut seropositivity, which could pose as an additional potential risk factor for horizontal HPV transmission [20, 28, 59, 91, 92]. A slightly higher seroprevalence with increasing numbers of siblings or household members was only found for low risk HPV types 6 and 11 in the fully adjusted models, which could be an indicator for hetero-inoculation due to horizontal transmission of HPV through genital warts, lesions in the oral cavity or laryngeal lesions among household members/siblings [3].

We observed a pronounced association of cutHPV seroprevalence with age. This result is in line with other studies, showing a strong increase of cutHPV in early years of childhood [13, 93]. There were no gender differences regarding cutaneous types, which was also expected as prevalence of skin warts is also not gender-specific [93, 94]. An association between ultraviolet radiation exposure and cutaneous HPV infection (mainly of the beta genus) was described in other studies as it may play a role in the development of basal cell carcinoma and squamous cell carcinoma of the skin [95–97]. However, we did not observe any statistical association between number of experienced sunburns and seropositivity of any cutHPV in our data.

Due to the wide range of different mucosal and cutaneous HPV types included in the assay, the determination of seroprevalence largely depends on the applied cutoff value. There is a variety of serological HPV antibody detection methods, a lack of a universal applicable reference, and no standard cutoff values for type-specific HPV serology. Therefore, serological HPV studies often use a most likely negative cohort as the negative reference to calculate cutoff values. This, however, assumes negativity in this cohort and is challenging if the ‘true’ status is unknown. Our data was based on previous used cutoffs, established in a cohort of South Korean young women who claimed to have never had sexual contacts before and used for other studies with the same serological assay before [41, 44]. However, the challenges of HPV serology methods implicates a limitation in the comparison and explanatory power of seroprevalence differences between studies using different methods. Despite this limitation, the huge sample size of our study allows to compare seroprevalence of different age groups among the children, as they were all tested with the same methods.

Another potential limitation is antibody cross-reactivity for phylogenetically closely related HPV types, which could lead to an overestimation of seroprevalence for some types (e.g., HPV-16 and -33). However, there is no substantial cross-reactivity across phylogenetic species (e.g., alpha 7 and alpha 9, i.e., HPV-16 and -18) or genera, so the majority of our results are expected to reflect type-specific results.

## Conclusion

To our knowledge, this is the first study on HPV antibody seroprevalence of cutaneous and mucosal HPV types among a large, representative sample of children and adolescents aged 1–17 years living in Germany. As a result of our study, we found varying age distributions in seroprevalence, dependent on type and tropism and we found low but non-zero seroprevalence for most tested mucHPV among children and adolescents. For HPV-16, only age and regional differences were associated factors with seropositivity. Compared to mucHPV, seroprevalence of cutHPV were higher and generally increased with age. Our study results provide population-based HPV seroprevalence data among children and adolescents and are an important data source of prior cumulative exposure to HPV in different age groups. Our data can serve as additional baseline data to understand the nature of HPV infections among children and adolescents and help evaluating the impact of the HPV vaccine introduction.

## Supplementary Information

**Additional file 1: Figure S1.** Selection criteria of HPV types.

**Additional file 2: Figure S2.** Percentile plot of the antibody reaction (MFI values) of muscosal HPV types HPV-6, HPV-11, HPV-16, HPV-18, HPV31, HPV33, HPV45, HPV52, HPV58, HPV seroprevalence study (n = 12,257, sera collected 2003–2006).

**Additional file 3: Figure S3.** Percentile plot of the antibody reaction (MFI values) of cutaneous HPV types HPV1, HPV4, HPV8, HPV10, HPV38, HPV41, HPV49, HPV seroprevalence study (n = 12,257, sera collected 2003–2006).

**Additional file 4: Figure S4.** Multiple HPV seropositivity by age, HPV seroprevalence study (n = 12,257, sera collected 2003–2006).

**Additional file 5: Table S1.** Seroprevalence of individual mucosal human papillomavirus types by gender and age, HPV seroprevalence study (n = 12,257, sera collected 2003–2006).

**Additional file 6: Table S2.** Seroprevalence of individual cutaneous human papillomavirus types by gender and age, HPV seroprevalence study (n = 12,257, sera collected 2003–2006).

**Additional file 7: Table S3.** Regression estimates for associated factors with seropositivity for HPV-6, HPV seroprevalence study (n = 12,257, sera collected 2003–2006).

**Additional file 8: Table S4.** Regression estimates for associated factors with seropositivity for HPV-11, HPV seroprevalence study (n = 12,257, sera collected 2003–2006).

**Additional file 9: Table S5.** Regression estimates for associated factors with seropositivity for HPV-18, HPV seroprevalence study (n = 12,257, sera collected 2003–2006).

## Data Availability

Data cannot be shared publicly because of confidentiality and personal data security restrictions. All data from the “German Health Interview and Examination Survey for Children and Adolescents (KiGGS)” are stored at the national public health institute Robert Koch-Institute. However, data are available upon request from the institutional data access for the scientific community as public use files: https://www.rki.de/EN/Content/Health_Monitoring/HealthSurveys/HealthSurveys_node.

## Abbreviations

CI: Confidence interval
cutHPV: Cutaneous HPV types
DKFZ: German Cancer Research Center
GNHIES98: German National Health Interview and Examination Survey 1998
HPV: Human papillomavirus
HPV-LR: Selection of low risk HPV types tested in our study: 6, 11
HPV-HR: Selection of high risk HPV types tested in our study: 16, 18, 31, 33, 45, 52, 58
HPV-cut: Selection of cutaneous HPV types tested in our study: 1, 4, 8, 10, 38, 41, 49
HPV-2val: HPV types in the bivalent vaccine: 16, 18
HPV-4val: HPV types in the quadrivalent vaccine: 6, 11, 16, 18
HPV-9val: HPV types in the nonavalent vaccine: 6, 11, 16, 18, 31, 33, 45, 52, 58
KiGGS: German Health Survey for Children and Adolescents
mucHPV: Mucoseal HPV types
RKI: Robert Koch-Institute
PR: Prevalence ratios
