# Supplementary figures and images for: Seroprevalence of mucosal and cutaneous human papillomavirus (HPV) types among children and adolescents in the general population in Germany
Source: BMC Infect Dis. 2022 Jan 10;22:44. doi: 10.1186/s12879-022-07028-8 (PMC8751243; doi:10.1186/s12879-022-07028-8)

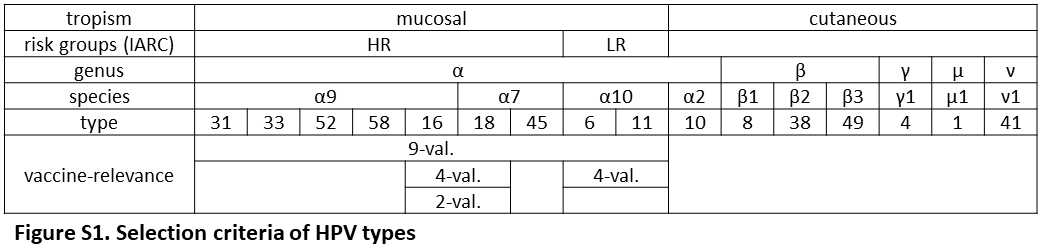

Supplement: Supplementary file 1 — Additional file 1: Figure S1. Selection criteria of HPV types. [file 12879_2022_7028_MOESM1_ESM.tif]

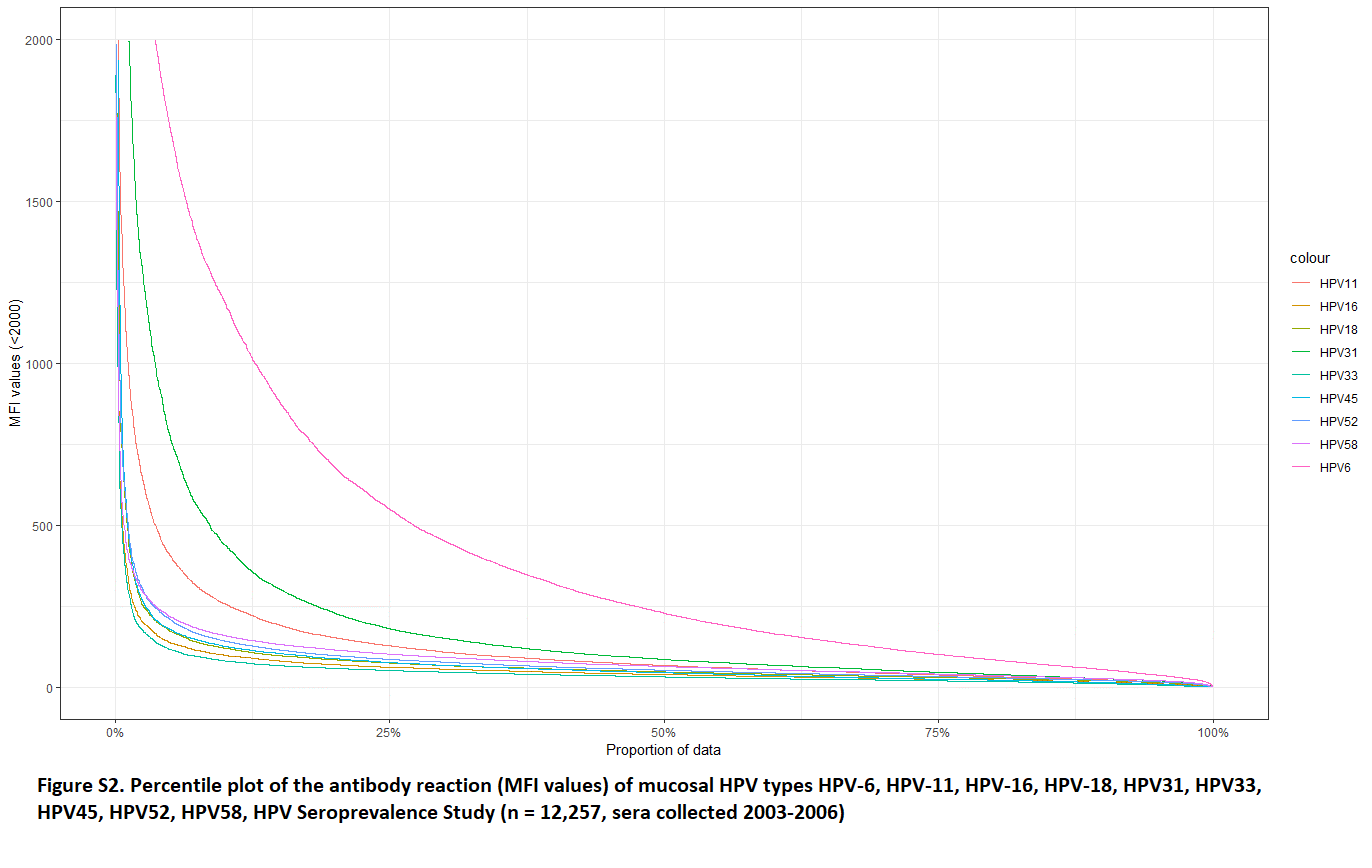

Supplement: Supplementary file 2 — Additional file 2: Figure S2. Percentile plot of the antibody reaction (MFI values) of muscosal HPV types HPV-6, HPV-11, HPV-16, HPV-18, HPV31, HPV33, HPV45, HPV52, HPV58, HPV seroprevalence study (n = 12,257, sera collected 2003–2006). [file 12879_2022_7028_MOESM2_ESM.tif]

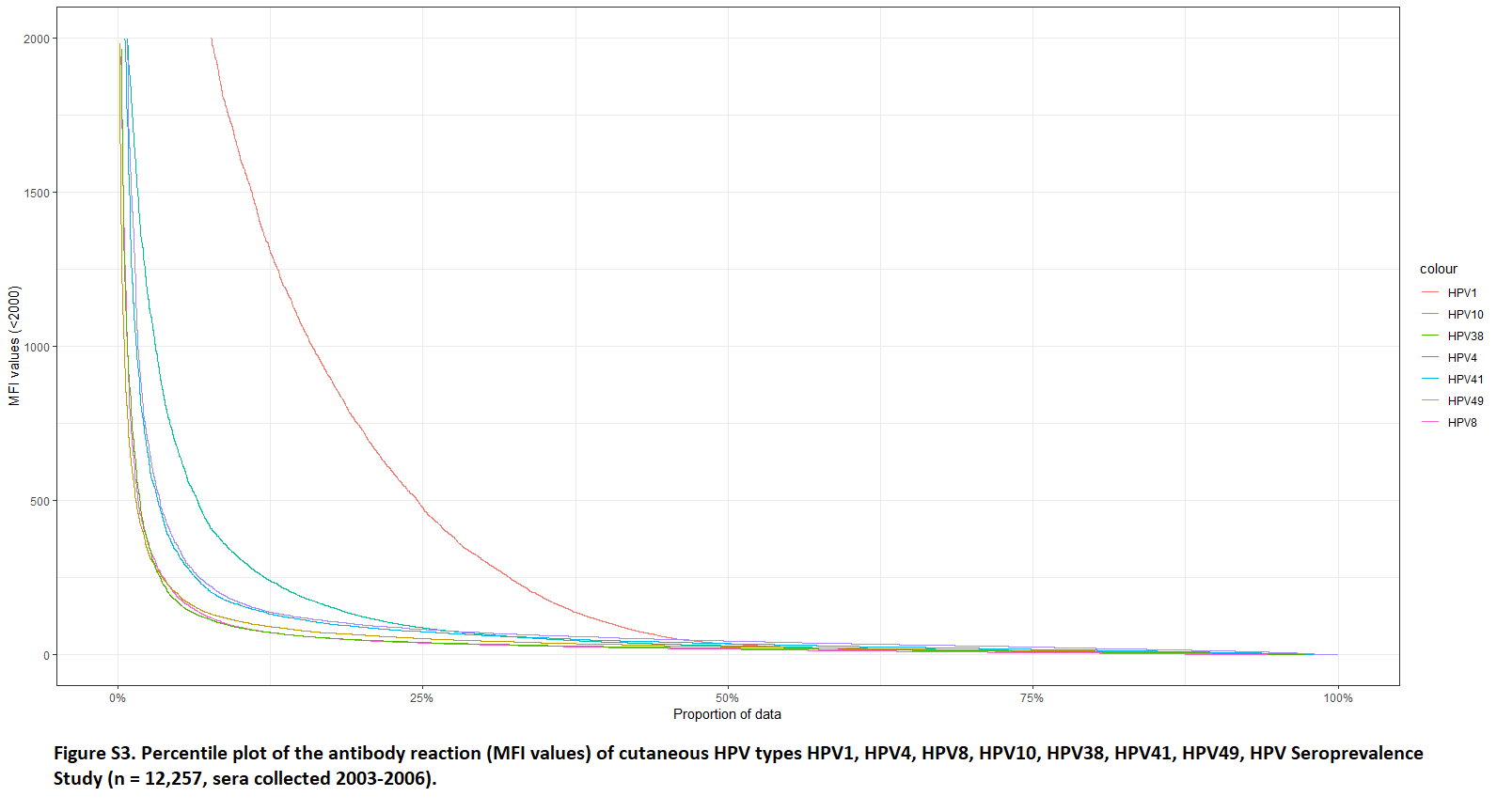

Supplement: Supplementary file 3 — Additional file 3: Figure S3. Percentile plot of the antibody reaction (MFI values) of cutaneous HPV types HPV1, HPV4, HPV8, HPV10, HPV38, HPV41, HPV49, HPV seroprevalence study (n = 12,257, sera collected 2003–2006). [file 12879_2022_7028_MOESM3_ESM.tif]

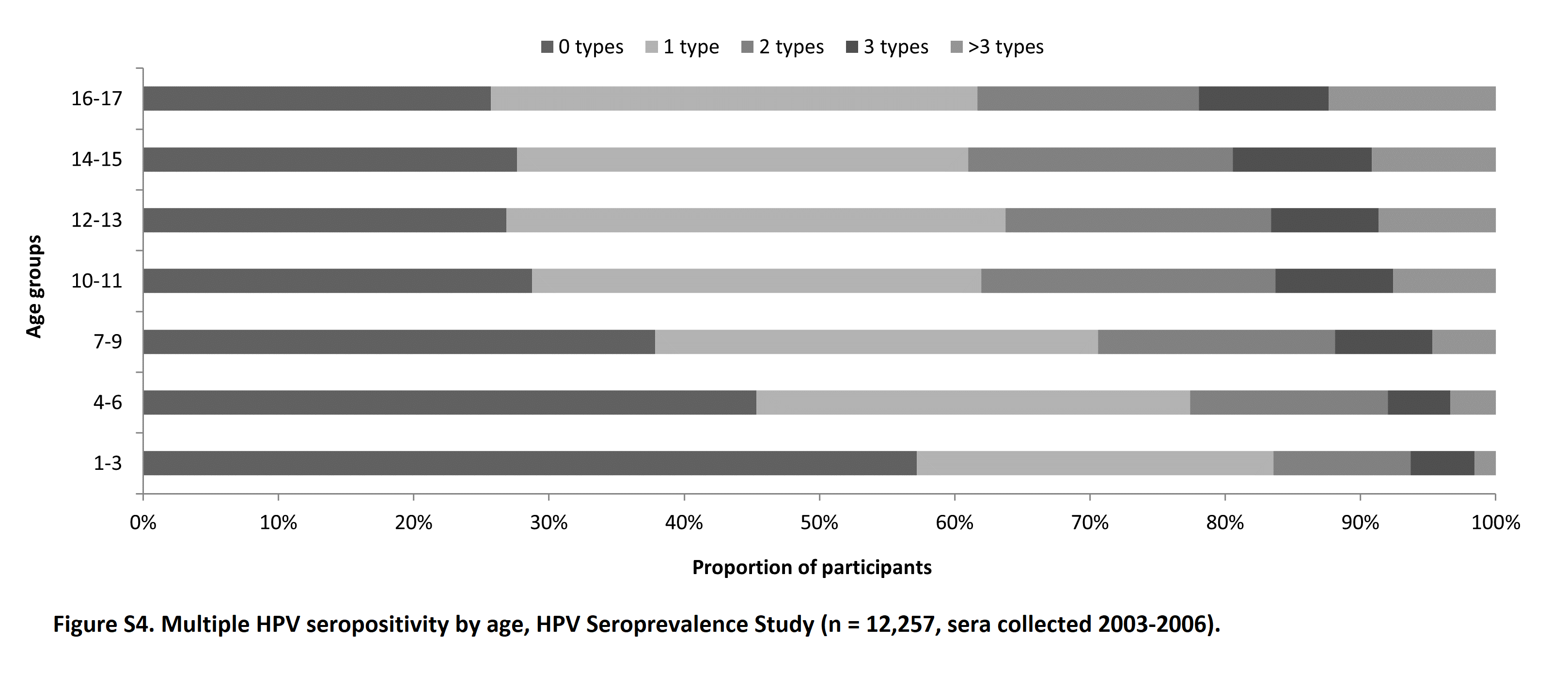

Supplement: Supplementary file 4 — Additional file 4: Figure S4. Multiple HPV seropositivity by age, HPV seroprevalence study (n = 12,257, sera collected 2003–2006). [file 12879_2022_7028_MOESM4_ESM.tif]
