# Supplementary material for: Seroprevalence of mucosal and cutaneous human papillomavirus (HPV) types among children and adolescents in the general population in Germany
Source: BMC Infect Dis. 2022 Jan 10;22:44. doi: 10.1186/s12879-022-07028-8 (PMC8751243; doi:10.1186/s12879-022-07028-8)
Supplement: Supplementary file 5 — Additional file 5: Table S1. Seroprevalence of individual mucosal human papillomavirus types by gender and age, HPV seroprevalence study (n = 12,257, sera collected 2003–2006). [file 12879_2022_7028_MOESM5_ESM.pdf]

Group	Subjects, no.	Seroprevalence by HPV type, % (95%CI)								
		6	11	16	18	31	33	45	52	58
Overall	12257	24.8 (23.6-26.1)	3.8 (3.3-4.4)	2.6 (2.2-3.0)	4.1 (3.5-4.7)	6.4 (5.8-7.1)	0.6 (0.4-0.8)	1.6 (1.4-1.9)	1.2 (1.0-1.5)	1.7 (1.4-2.1)
		2944	445	309	465	686	68	202	127	181
Females	5973	23.1 (21.5-24.7)	3.8 (3.2-4.5)	2.5 (2.0-3.1)	3.7 (3.0-4.4)	6.4 (5.5-7.4)	0.7 (0.4-1.0)	1.6 (1.2-2.0)	1.2 (0.9-1.6)	1.7 (1.3-2.3)
Age groups		1302	205	150	222	337	34	105	61	90
1-3	615	18.4 (14.7-22.8)	2.7 (1.6-4.6)	1.5 (0.8-2.8)	4.0 (2.4-6.4)	14.4 (11.0-18.6)	1.1 (0.4-3.0)	0.9 (0.4-2.2)	0.3 (0.1-1.1)	0.9 (0.3-2.4)
		115	17	11	24	85	6	7	3	5
4-6	882	31.6 (27.5-36.0)	6.4 (4.4-9.1)	2.1 (1.2-3.6)	2.6 (1.6-4.0)	7.6 (5.8-9.8)	0.7 (0.3-1.9)	1.2 (0.6-2.6)	0.9 (0.4-2.2)	1.7 (1.0-3.0)
		246	47	16	25	68	6	10	8	17
7-9	1071	28.1 (24.9-31.5)	5.1 (3.7-6.9)	2.2 (1.2-4.2)	3.6 (2.5-5.1)	5.7 (4.1-7.7)	0.2 (0.1-0.6)	1.0 (0.5-2.0)	0.9 (0.4-2.0)	1.2 (0.6-2.3)
		296	50	16	37	60	6	13	10	11
10-11	823	26.2 (22.4-30.5)	4.4 (2.9-6.8)	2.7 (1.7-4.2)	3.7 (2.3-5.9)	5.0 (3.5-7.0)	0.3 (0.1-1.3)	1.8 (0.9-3.6)	1.5 (0.6-3.8)	1.1 (0.5-2.3)
		201	34	22	27	39	2	12	5	7
12-13	889	20.3 (17.1-23.9)	2.5 (1.5-4.2)	2.1 (1.2-3.7)	4.4 (3.1-6.4)	3.4 (2.2-5.2)	0.8 (0.3-1.9)	2.5 (1.4-4.2)	1.2 (0.6-2.3)	2.6 (1.4-4.9)
		167	23	20	35	30	5	20	9	17
14-15	858	17.4 (14.6-20.7)	1.8 (1.0-3.2)	3.7 (2.4-5.6)	3.6 (2.5-5.1)	3.0 (1.8-4.7)	0.0 (0.0-0.0)	1.8 (1.1-2.8)	0.6 (0.2-1.5)	2.0 (0.9-4.3)
		142	16	31	35	23	0	20	5	14
16-17	834	17.1 (14.3-20.3)	2.9 (1.7-4.7)	3.9 (2.7-5.6)	4.1 (2.9-5.9)	4.0 (2.6-6.3)	1.4 (0.7-2.8)	2.4 (1.5-3.7)	3.2 (1.9-5.4)	2.5 (1.4-4.3)
		134	18	34	39	31	9	23	20	18
Males	6284	26.4 (24.9-28.0)	3.8 (3.2-4.5)	2.7 (2.2-3.2)	4.5 (3.7-5.3)	6.4 (5.5-7.5)	0.6 (0.4-0.8)	1.7 (1.3-2.1)	1.2 (0.8-1.7)	1.7 (1.3-2.3)
Age groups		1642	240	159	243	349	34	97	66	91
1-3	649	19.3 (15.7-23.5)	4.2 (2.6-6.8)	1.6 (0.7-3.3)	4.7 (3.0-7.3)	15.5 (12.3-19.3)	0.9 (0.3-2.7)	0.4 (0.1-1.1)	1.0 (0.3-3.3)	2.1 (0.9-4.6)
		141	28	10	27	99	6	4	3	7
4-6	933	35.8 (32.2-39.5)	4.9 (3.5-6.8)	2.6 (1.6-4.0)	4.7 (3.0-7.2)	8.1 (6.1-10.9)	0.2 (0.1-0.8)	1.9 (1.0-3.5)	1.5 (0.7-3.2)	1.8 (0.9-3.4)
		338	49	27	34	66	2	11	11	12
7-9	1146	33.9 (30.4-37.5)	4.9 (3.6-6.6)	2.7 (1.6-4.5)	4.4 (3.2-6.1)	4.6 (3.4-6.3)	0.9 (0.4-1.9)	2.1 (1.2-3.5)	1.3 (0.7-2.7)	1.4 (0.8-2.4)
		382	62	29	44	58	8	20	14	17
10-11	851	33.4 (29.4-37.7)	4.4 (3.0-6.4)	2.1 (1.1-4.0)	2.6 (1.5-4.8)	3.1 (1.9-4.9)	0.1 (0.0-0.6)	1.2 (0.5-2.7)	0.8 (0.4-2.0)	1.3 (0.5-3.1)
		257	34	15	19	28	1	8	7	9
12-13	960	23.4 (20.3-26.8)	3.1 (1.9-5.0)	2.1 (1.3-3.4)	3.6 (2.4-5.3)	4.4 (3.1-6.1)	0.5 (0.2-1.3)	1.7 (0.9-3.2)	1.2 (0.7-2.2)	1.4 (0.7-2.6)
		209	24	22	37	38	4	17	12	14
14-15	963	20.8 (17.9-24.1)	3.1 (1.9-5.1)	2.5 (1.6-3.9)	4.7 (3.2-6.8)	4.4 (2.8-6.6)	0.6 (0.3-1.3)	1.6 (0.9-2.9)	1.4 (0.7-2.9)	2.1 (1.2-3.8)
		196	26	25	44	33	7	19	12	17
16-17	782	16.2 (13.1-19.9)	1.9 (1.1-3.2)	5.0 (3.4-7.3)	6.0 (4.3-8.4)	3.2 (2.1-5.0)	0.6 (0.3-1.4)	2.8 (1.5-5.0)	0.9 (0.4-2.3)	2.0 (1.1-3.7)
		119	17	31	38	27	6	18	7	15

Table S1. Seroprevalence of Individual Mucosal Human Papillomavirus Types by Gender and Age, HPV Seroprevalence Study (n = 12,257, sera collected 2003-2006). NOTE. CI, confidence interval.
