# Supplementary material for: Seroprevalence of mucosal and cutaneous human papillomavirus (HPV) types among children and adolescents in the general population in Germany
Source: BMC Infect Dis. 2022 Jan 10;22:44. doi: 10.1186/s12879-022-07028-8 (PMC8751243; doi:10.1186/s12879-022-07028-8)
Supplement: Supplementary file 6 — Additional file 6: Table S2. Seroprevalence of individual cutaneous human papillomavirus types by gender and age, HPV seroprevalence study (n = 12,257, sera collected 2003–2006). [file 12879_2022_7028_MOESM6_ESM.pdf]

Group	Subjects, no.	Seroprevalence by HPV type, % (95%CI)						
		1	4	8	10	38	41	49
Overall	12257	31.7 (30.6-32.8)	13.6 (12.8-14.4)	4.5 (4.1-5.0)	5.0 (4.5-5.6)	4.0 (3.6-4.4)	7.5 (6.8-8.3)	8.1 (7.3-9.0)
		4181	1757	563	591	504	943	1022
Females	5973	33.8 (32.2-35.5)	13.7 (12.7-14.8)	4.3 (3.7-4.9)	5.3 (4.6-6.1)	4.0 (3.5-4.6)	8.0 (7.1-9.0)	7.9 (6.9-8.9)
Age, years		2162	871	284	291	246	487	499
1-3	615	2.1 (1.1-4.0)	5.4 (3.5-8.4)	1.5 (0.6-3.7)	3.5 (1.9-6.2)	2.0 (1.0-4.0)	2.2 (1.2-3.9)	1.7 (0.9-2.9)
		10	31	6	18	11	15	15
4-6	882	10.6 (8.1-13.7)	6.6 (4.8-9.0)	1.6 (0.9-2.6)	4.1 (2.6-6.6)	2.5 (1.6-3.8)	4.0 (2.7-5.8)	3.6 (2.3-5.6)
		81	61	19	31	23	37	30
7-9	1071	29.3 (25.6-33.2)	11.5 (9.1-14.4)	2.4 (1.6-3.5)	5.9 (4.3-8.1)	3.9 (2.8-5.4)	5.4 (4.0-7.2)	6.0 (4.4-8.2)
		315	114	32	53	41	59	64
10-11	823	50.5 (46.4-54.6)	13.6 (10.7-17.1)	3.0 (2.0-4.5)	4.6 (3.0-6.8)	3.1 (1.9-5.1)	9.4 (7.1-12.4)	7.7 (5.6-10.4)
		396	117	31	35	22	63	64
12-13	889	51.2 (46.6-55.7)	18.5 (15.7-21.7)	4.7 (3.4-6.5)	6.0 (4.0-8.7)	4.1 (2.9-5.8)	10.3 (7.9-13.4)	11.8 (9.7-14.2)
		450	162	46	51	38	89	108
14-15	858	51.9 (48.0-55.9)	21.5 (18.5-24.7)	8.0 (6.0-10.4)	6.4 (4.6-8.8)	6.2 (4.5-8.4)	12.5 (10.2-15.3)	11.7 (9.3-14.6)
		445	188	66	49	50	98	94
16-17	834	55.7 (51.7-59.6)	22.6 (19.8-25.8)	9.9 (7.3-13.3)	6.7 (4.8-9.1)	6.9 (5.3-9.1)	14.8 (12.1-18.1)	15.2 (12.2-18.8)
		465	198	84	54	61	126	124
Males	6284	29.7 (28.4-31.1)	13.5 (12.4-14.6)	4.7 (4.1-5.4)	4.8 (4.1-5.6)	4.0 (3.5-4.5)	7.1 (6.2-8.1)	8.4 (7.3-9.5)
Age, years		2019	886	279	300	258	456	523
1-3	649	0.9 (0.4-1.8)	6.0 (3.9-9.3)	1.5 (0.7-3.1)	5.0 (3.0-8.1)	1.9 (1.0-3.8)	4.3 (2.7-6.9)	2.6 (1.4-4.7)
		9	32	9	24	14	25	17
4-6	933	10.7 (8.4-13.6)	4.9 (3.6-6.8)	3.8 (2.5-5.9)	2.7 (1.8-4.0)	3.4 (2.2-5.2)	2.8 (1.9-4.2)	6.8 (4.7-9.7)
		81	52	28	32	27	32	48
7-9	1146	27.3 (24.4-30.3)	7.2 (5.7-9.0)	3.0 (2.1-4.4)	3.1 (2.1-4.6)	2.9 (2.0-4.0)	5.3 (3.7-7.6)	6.3 (4.7-8.4)
		301	98	35	39	42	57	72
10-11	851	40.0 (35.9-44.2)	17.3 (14.2-20.9)	5.1 (3.5-7.3)	6.4 (4.5-9.0)	5.2 (3.7-7.2)	7.5 (5.7-9.7)	9.7 (7.5-12.5)
		346	128	39	44	41	71	86
12-13	960	46.8 (42.5-51.2)	17.7 (14.7-21.2)	5.5 (4.1-7.4)	5.7 (4.0-8.1)	5.3 (3.7-7.7)	8.6 (6.6-11.2)	9.9 (7.6-12.7)
		451	160	50	52	43	79	95
14-15	963	46.6 (42.5-50.7)	22.8 (19.8-26.1)	5.6 (4.1-7.6)	6.7 (5.0-8.7)	4.4 (3.1-6.1)	10.4 (8.4-12.8)	11.8 (9.4-14.8)
		456	212	50	67	43	99	106
16-17	782	47.7 (43.4-52.0)	23.8 (20.7-27.1)	9.5 (7.3-12.2)	5.2 (3.7-7.3)	5.6 (4.0-7.8)	12.6 (9.9-15.9)	13.4 (10.8-16.5)
		375	204	68	42	48	93	99

Table S2. Seroprevalence of Individual Cutaneous Human Papillomavirus Types by Gender and Age, HPV Seroprevalence Study (n = 12,257, sera collected 2003-2006). NOTE. CI, confidence interval.
