# Supplementary material for: Seroprevalence of mucosal and cutaneous human papillomavirus (HPV) types among children and adolescents in the general population in Germany
Source: BMC Infect Dis. 2022 Jan 10;22:44. doi: 10.1186/s12879-022-07028-8 (PMC8751243; doi:10.1186/s12879-022-07028-8)
Supplement: Supplementary file 7 — Additional file 7: Table S3. Regression estimates for associated factors with seropositivity for HPV-6, HPV seroprevalence study (n = 12,257, sera collected 2003–2006). [file 12879_2022_7028_MOESM7_ESM.pdf]

| | Crude PR (95%CI) | p-value | Fully adjusted PR (95%CI)$ | p-value |
|---|---|---|---|---|
| Gender | | | | |
| Female | Ref | | Ref | |
| Male | 1.1 (1.1-1.2) | 0.001 | 1.2 (1.1-1.3) | <0.001 |
| Age group (years) | | | | |
| 1-3 | Ref | | Ref | |
| 4-6 | 1.8 (1.5-2.1) | <0.001 | 1.7 (1.4-2.0) | <0.001 |
| 7-9 | 1.6 (1.4-1.9) | <0.001 | 1.5 (1.3-1.8) | <0.001 |
| 10-11 | 1.6 (1.3-1.9) | <0.001 | 1.5 (1.2-1.7) | <0.001 |
| 12-13 | 1.2 (1.0-1.4) | 0.133 | 1.1 (0.9-1.3) | 0.604 |
| 14-15 | 1.0 (0.8-1.2) | 0.910 | 0.9 (0.8-1.1) | 0.514 |
| 16-17 | 0.9 (0.7-1.1) | 0.199 | 0.8 (0.7-1.0) | 0.105 |
| Region of Residence | | | | |
| West Germany | Ref | | Ref | |
| East Germany | 0.8 (0.7-0.9) | <0.001 | 0.8 (0.7-0.9) | 0.001 |
| Urbanity | | | | |
| Rural | Ref | | Ref | |
| Small City | 1.2 (1.0-1.4) | 0.041 | 1.1 (1.0-1.3) | 0.107 |
| Medium Sized City | 1.1 (0.9-1.3) | 0.211 | 1.1 (0.9-1.2) | 0.446 |
| Large City | 1.2 (1.0-1.4) | 0.037 | 1.2 (1.0-1.4) | 0.038 |
| Socioeconomic status of parents | | | ns# | |
| Low | Ref | | | |
| Middle | 1.0 (0.9-1.1) | 0.448 | | |
| High | 1.0 (0.9-1.1) | 0.625 | | |
| Migratory background of parents | | | ns# | |
| None | Ref | | | |
| One parent | 1.1 (1.0-1.3) | 0.085 | | |
| Both parents | 1.2 (1.0-1.3) | 0.005 | | |
| Number of household members | | | ns# | |
| | 1.1 (1.1-1.1) | <0.001 | | |
| Number of siblings in household | | | | |
| | 1.1 (1.1-1.1) | <0.001 | 1.1 (1.1-1.1) | <0.001 |
| BMI | | | ns# | |
| | 1.0 (1.0-1.0) | <0.001 | | |

**Table S3. Regression estimates for associated factors with seropositivity for HPV-6, HPV Seroprevalence Study (n = 12,257, sera collected 2003-2006).** NOTES. PR Prevalence Ratio, CI Confidence Interval, Ref Reference, $ Mutually adjusted for all other variables in the model, #ns= Variables were not significantly associated with HPV seroprevalence in the final model and therefore excluded
