# Supplementary material for: Seroprevalence of mucosal and cutaneous human papillomavirus (HPV) types among children and adolescents in the general population in Germany
Source: BMC Infect Dis. 2022 Jan 10;22:44. doi: 10.1186/s12879-022-07028-8 (PMC8751243; doi:10.1186/s12879-022-07028-8)
Supplement: Supplementary file 8 — Additional file 8: Table S4. Regression estimates for associated factors with seropositivity for HPV-11, HPV seroprevalence study (n = 12,257, sera collected 2003–2006). [file 12879_2022_7028_MOESM8_ESM.pdf]

	Crude PR (95%CI)	p-value	Fully adjusted PR (95%CI)$	p-value
Gender				
Female	Ref			
Male	1.0 (0.8-1.3)	0.879	ns#	
Age group (years)				
1-3	Ref		Ref	
4-6	1.6 (1.1-2.4)	0.021	1.6 (1.0-2.4)	0.030
7-9	1.4 (1.0-2.1)	0.072	1.4 (0.9-2.0)	0.111
10-11	1.3 (0.8-2.0)	0.318	1.2 (0.7-1.9)	0.502
12-13	0.8 (0.5-1.3)	0.372	0.8 (0.5-1.2)	0.262
14-15	0.7 (0.4-1.2)	0.201	0.7 (0.4-1.2)	0.161
16-17	0.7 (0.4-1.1)	0.141	0.7 (0.4-1.2)	0.189
Region of Residence				
West Germany	Ref		Ref	
East Germany	0.6 (0.5-0.9)	0.007	0.7 (0.5-0.9)	0.021
Urbanity			ns#	
Rural	Ref			
Small City	1.4 (0.9-2.1)	0.166		
Medium Sized City	1.2 (0.7-1.8)	0.542		
Large City	1.6 (1.0-2.5)	0.058		
Socioeconomic status of parents			ns#	
Low	Ref			
Middle	1.1 (0.9-1.5)	0.371		
High	1.2 (0.9-1.6)	0.270		
Migratory background of parents			ns#	
None	Ref			
One parent	0.9 (0.6-1.5)	0.674		
Both parents	1.1 (0.9-1.5)	0.405		
Number of household members				
	1.1 (1.1-1.2)	0.002	1.1 (1.0-1.2)	0.043
Number of siblings in household			ns#	
	1.1 (1.0-1.2)	0.053		
BMI			ns#	
	1.0 (0.9-1.0)	0.004		

Table S4. Regression estimates for associated factors with seropositivity for HPV-11, HPV Seroprevalence Study (n = 12,257, sera collected 2003-2006). NOTES. PR Prevalence Ratio, CI Confidence Interval, Ref Reference, $ Mutually adjusted for all other variables in the model, #ns= Variables were not significantly associated with HPV seroprevalence in the final model and therefore excluded
