# Supplementary material for: Seroprevalence of mucosal and cutaneous human papillomavirus (HPV) types among children and adolescents in the general population in Germany
Source: BMC Infect Dis. 2022 Jan 10;22:44. doi: 10.1186/s12879-022-07028-8 (PMC8751243; doi:10.1186/s12879-022-07028-8)
Supplement: Supplementary file 9 — Additional file 9: Table S5. Regression estimates for associated factors with seropositivity for HPV-18, HPV seroprevalence study (n = 12,257, sera collected 2003–2006). [file 12879_2022_7028_MOESM9_ESM.pdf]

	Crude PR (95%CI)	p-value	Fully adjusted PR (95%CI)$	p-value
Gender			ns#	
Female				
Male	1.2 (1.0-1.5)	0.095		
Age group (years)			ns#	
1-3	Ref			
4-6	0.8 (0.5-1.3)	0.463		
7-9	0.9 (0.6-1.3)	0.650		
10-11	0.7 (0.5-1.2)	0.182		
12-13	0.9 (0.6-1.4)	0.707		
14-15	1.0 (0.7-1.4)	0.800		
16-17	1.2 (0.8-1.7)	0.421		
Region of Residence				
West Germany	Ref		Ref	
East Germany	0.7 (0.5-1.0)	0.055	0.7 (0.5-1.0)	0.033
Urbanity				
Rural	Ref		Ref	
Small City	0.9 (0.6-1.2)	0.466	0.8 (0.6-1.2)	0.320
Medium Sized City	0.7 (0.5-1.0)	0.069	0.7 (0.4-1.0)	0.041
Large City	0.9 (0.6-1.3)	0.421	0.8 (0.6-1.2)	0.381
Socioeconomic status of parents			ns#	
Low	Ref			
Middle	1.1 (0.9-1.5)	0.359		
High	0.9 (0.7-1.2)	0.407		
Migratory background of parents			ns#	
None	Ref			
One parent	1.2 (0.8-1.8)	0.451		
Both parents	0.9 (0.6-1.3)	0.523		
Number of household members			ns#	
	1.0 (0.9-1.1)	0.985		
Number of siblings in household			ns#	
	1.0 (0.9-1.2)	0.707		
BMI			ns#	
	1.0 (1.0-1.0)	0.399		

Table S5. Regression estimates for associated factors with seropositivity for HPV-18, HPV Seroprevalence Study (n = 12,257, sera collected 2003-2006). NOTES. PR Prevalence Ratio, CI Confidence Interval, Ref Reference, $ Mutually adjusted for all other variables in the model, #ns= Variables were not significantly associated with HPV seroprevalence in the final model and therefore excluded
